# St. George's Hospital

**Published:** 1829-07-01

**Authors:** 


### XVIII.

### ST. GEORGE'S HOSPITAL.

### I. CASES IN WHICH THE BLOOD WAS FOUND AFTER DEATH PRETERNATURALLY FLUID.

The life or death, the innocence or guilt of a fellow creature, having recently been made, we might almost say, to hinge on the fluid condition of the blood after death, in the body of Mr. Neale, we gladly seize on the opportunity offered us of shewing how fallacious a criterion of violence inflicted this particular condition of the blood must be, by the mention of cases that occurred at St. George's Hospital, in which it was present in a more or less marked degree. This fluid state of the blood is really far from unfrequent in post-mortem examinations, and doubtless were it oftener looked for, it would oftener be found than even at present it is. There are certain diseases, in which, on the whole, it is noticed with more regularity and constancy than in others, and of these we think that phlebitis may be ranked as the head and front. We know that many cases of fatal inflammation of the veins are on record, where no such condition of the blood is alluded to, and consequently where, in all probability, it did not exist. These, however, are the exceptions rather than the rule, and we must confess that, in the cases we ourselves have been fortunate or unfortunate enough to witness, the phenomenon in question was almost invariably present.

The following, which occurred upwards of a year ago, was a very well marked instance of the fact.

Case 1. *Inflammation of the Cutaneous Veins of the Leg and Thigh—Death —Non-coagulation of the Blood.*

A butler, 56 years of age, was admitted into hospital October 23d, 1827, under the care of Mr. Brodie, with varices of the veins of the right leg and foot, and a small varicose ulcer above the internal malleolus. Under the use of poultice and soap-strappings, &c. he went on tolerably well, till the morning of the 15th of November, when he had a sharp rigor, followed during the day by general malaise, and pain in the course of the veins during the night. On the 16th, the thigh was found to be swollen, and the vein evidently inflamed. The swelling of the limb and inflammation of the vein increased, delirium and rapid prostration succeeded, mortification, or rather gangrene, was established in the limb, and at 2, p. m. of the 17th, the patient died.

On dissection, the coats of the inflamed vein were a good deal thickened, its cavity filled with loose, broken down coagulum, and its inner membrane of a dark red colour. This staining of the internal coat was traced along the great venous trunks to the heart, which was soft and flabby in its texture, and *distended with dark fluid blood,* having only one or two very trifling portions of coagulum mixed with it. The aorta, and indeed the arterial system generally, was highly coloured, not in patches, but with one uniform and vivid red, not extending, however, beyond the inner coat. The blood throughout the body was uncoagulated, and the veins upon the surface could be traced by their purple colour, almost as distinctly as if they had been injected. The staining of the inner coat of the aorta was so marked, that the vessel was removed from the body on that account, and is or was in Mr. Brodie's museum at the School of Great Windmill Street.

Traumatic gangrene, and likewise, we suppose, the other varieties of gangrene not traumatic, frequently present this appearance of the circulating fluid.

Case 2. *Compound Fracture of the Right Leg—Sloughing of the Limb—Amputation—Death—Staining of the Femoral Vessels.*

James Scarlett, ætatis 57, was admitted December 1st, under the care of Mr. Brodie, in consequence of a compound fracture of both bones of the right leg about three inches above the malleolus, received by falling from a scaffold, 20 feet high, in the works going on at Apsley House. He went on tolerably well till the evening of the next day, when he became anxious and low, and during that night was delirious. On the 3d, complete sloughing of the subcutaneous cellular membrane, with commencing death of the integuments had taken place from the instep to the knee. The system, as might be expected under such circumstances, was extremely depressed, and, seeing that if nothing were done the man must inevitably die, Mr. Brodie removed the limb, some three or four inches above the knee. He slept for an hour after the operation, and appeared somewhat better in the evening, but the calm was delusive—he became affected with delirium and vomiting in the night—the symptoms of sinking soom became established—and, after lingering till the 6th, he died on that day at 2, p. m.

*Dissection.* Passing over some particulars foreign to our present purpose, we may observe, that on cutting down to the femoral vessels in the groin, and following them on from that to the face of the stump, they presented no unusual appearance from without. On being removed from the limb and laid open, both artery and vein were found to be more or less filled with dark and semi-fluid blood, and both presented on their internal coats a deep and nearly a blood-red dye. This was very far from the *injected* appearance caused by inflammation, but evidently a *staining* merely of the coats in contact with the semi-fluid blood. The lining membrane of the heart, especially the right chambers, of the venæ cavæ, and even of the aorta, was more or less tinged by the blood, which in all was fluid, or nearly so.

We could give some further instances of this unnaturally fluid condition of the blood in phlebitis, gangrene, and some of those cases of purulent depôts, so often met with in hospital practice. However, it would be tedious to ourselves and to our readers to multiply examples of the fact; suffice it to say that such it is. The case we are next to describe was of a different character from any of the preceding, and has already been noticed, with another view, in our pages.

CASE 3. *Removal of part of a thickened Prepuce—Insidious and fatal Pneumonia—Fluid State of the Blood.*

A young man, of four or five and twenty years of age, was admitted under the care of Mr. Keate, on the 18th of February last, with the prepuce much thickened after paraphymosis. The thickened part was removed by Mr. K. on the 20th, and on the 22d the patient was observed to be nervous and irritable to an extraordinary degree. On the 23d, he was in a state of great depression, with hurried breathing, but no cough, and perfect power of taking a full inspiration. At twelve o'clock next day he died.

*Dissection.* On raising the sternum, the veins passing to the right side of the heart were gorged with black and fluid blood. The right chambers of the heart were also gorged with fluid blood, and the left were much more filled with the same than they usually are; so much indeed was this the case, that it became a matter of remark and dispute on its cause in the dead-house. On removing the calvarium, the veins of the dura and pia mater were turgid, and filled with more or less fluid blood, whilst the section of the brain displayed numerous and large *black* points, evidently the dark venous blood contained in its vessels. The lungs we may mention were violently inflamed, the pleuræ too inflamed, but in a less degree.

On the 2d or 3d of the present April, a female patient of Dr. Seymour's who had laboured for some years under Winter cough, and died, we believe, with some symptoms of thoracic inflammation, was examined in the hospital dead-house, and found to present the fluidity of the blood to a very great degree. The gentleman, Mr. Good, who conducted the examination, informed us that scarcely a portion of coagulum could be found in the heart or veins, for he opened many in the body. The mitral valve in this subject was affected with partial ossification, and the lining membrane of the bronchi was much inflamed. Still more lately a corpse presented this phenomenon, conjoined with ulcerations of the mucous membrane of the intestines, near the junction of the ileum and cæcum.

We think that the cases here brought forward would suffice of themselves to shake the faith of any unprejudiced man, in the value of the fluidity of the blood as a test of this or that state of system prior to the fatal event, and especially as a medico-legal test of violence inflicted. It may, and no doubt it very commonly is, a concomitant or consequence of sudden death, but it certainly is also found when the death is slow and produced by ordinary causes, in the ordinary way. The why and the wherefore we do not understand, most probably we never shall; but the fact is as certain as that two and two make four, and sorry we should be to fling it in the balance against a prisoner, when Justice is weighing his guilt or innocence.

II. CURIOUS MORBID CONDITION OF THE ARACHNOID MEMBRANE OF THE BRAIN.

There is an appearance presented by the arachnoid membrane, which is not unfrequently met with in post-mortem investigation, which looks very like the effects of disease, and is often, no doubt, considered as such, but which, we are strongly inclined to suspect, is not of the nature it would seem to be. The first case in which we particularly observed it, was that of the young man who died of peripneumony after the removal of part of his prepuce, already more than once referred to. The patient in question had, by all accounts, been healthy, had never presented any "head symptoms," as far as we know or could learn, and died of inflammation in a distant organ—the tissue of the lungs. The second case that fell under our notice, was that of a diabetic, under the care of Dr. Hewett, who died, we believe, (for we did not see him during life,) with some curious symptoms after, or rather during the exhibition of opium. Perhaps the details of this dissection may not be uninteresting, and, at all events, will serve to explain the particular lesion, if such it can be called, of the arachnoid tunic.

The body was that of a man beyond the middle term of life, not so very much attenuated, but generally bloodless. The lungs were as healthy as lungs could be; the heart was extremely flabby, the right ventricle being scarcely thicker in its walls than pasteboard, and the left proportionably weak. The kidneys were pale in colour, and were said to present in some parts points of ecchymosis, though we cannot say that we saw them ourselves.

The tunica arachnoides had put on a peculiar semi-opaque appearance, easily recognized when once seen, but difficult to describe. It seemed rather to depend on a slight fluid effusion between the arachnoid and pia mater, than on any actual structural alteration of either membrane. It was generally over the upper surface of the brain, but scarcely if at all perceived at the basis, which is not the case as far as we have seen, in true inflammation of the cerebral meninges. At one or two points, between the clefts of the convolutions, the opacity was decided, and appeared to depend upon solid lymph, which, however, on making the section of the part, was not the case. One or two slices of the upper surface of the hemispheres had been put aside, to preserve, or attempt to preserve, the appearance, but, on looking at them shortly afterwards, it had almost entirely vanished, evidently from the fluid having escaped where the sections had been made.

Another very well-marked instance of this condition was afforded by a patient of Dr. Seymour's, who had laboured long, and, in fact, died from, the effects of the emanations of lead. This patient had had epileptic fits. Still more recently, we have seen it in the body of a woman who died with jaundice and hemiplegia. Here, again, the dissection was one of some interest, and, therefore, we shall give it at large.

The body, externally, was very yellow, of course. The conjunctivæ were deeply tinged, and the clear part of the eye had also a light shade of yellow; but, whether that depended on the colouring of the conjunctival prolongation over the cornea, the cornea itself, or the aqueous humour, is really more than we can venture to say. Nothing unusual was discovered in the chest. On opening the abdomen, the duodenum, biliary ducts, and liver were removed, but no mechanical obstruction to the flow of the bile could be found. The bile itself was of a darkish olive-green, and slightly viscid. The liver was unhealthy, and, towards the back part of its right lobe, was an abscess in its parenchyma. The spleen, both externally and internally, was most unequivocally tinged. One or more round concretions, the size of small peas, were found in the spermatic veins.

The pericranium and bones of the skull were remarkably yellow, and the former very easily separable from the latter. The mucus in the frontal sinus was discoloured. A kind of fibrous appearance was noticed on the dura mater, and the arachnoid membrane presented the same appearance as the other cases already described, an appearance depending in this, as in them, more on a fluid effusion beneath the membrane, than any alteration of its own texture. One remarkable difference between this and the preceding instances was, that, instead of being white, the opacity was yellow, in accordance with the general discoloration of the body. On cutting off a portion of the brain and setting it aside, this opacity passed away in some degree. The cortical part of the brain had, we thought, a slight tinge, but the tint, if such it was, of the medullary portion, was still less decisive. There were three or four drachms of yellow serum in the ventricles. In one of the hemispheres of the cerebellum, (on the side opposite to that which had been palsied) was a kind of sloughy cavity, containing, not pus, but the matter of the cerebellum, in a state of ramollissement, one might almost say putridity. This disorganization was pretty extensive.

We have deemed it right to describe this condition of the arachnoid membrane, and detail the cases in which we have observed it, because we consider the subject one of interest. What the cause of this effusion beneath the membrane may be, we leave to those who like to explain. Is it to be considered as the evidence of old and chronic inflammation? For our own parts, we think not; though we readily allow, that the few cases in which we have remarked it are far, very far, indeed, from settling the question one way or the other. It may easily be objected to the non-inflammatory view of the phenomenon, that, in three of the cases out of the four, there was either mischief going on in the head, or disordered action set up in it. The diabetic died under the influence of opium, we do not say *of* it; the painter had suffered from epilepsy; and the jaundiced patient had hemiplegia, and unequivocal disorganization in the cerebellum. Upon the whole, then, we must leave the point undecided, and open to further observation, which, in our opinion, it really deserves.

### XXXVIII.

### ST. GEORGE'S HOSPITAL.

### I. OPEN FORAMEN OVALE.

*Case* 1. A very fine little girl, four or five years old, was run over by the state carriage of the Princess Sophia of Gloucester, on the 30th of April, the day of the Drawing Room at St. James's. Both wheels passed over the body of the child, who it seemed, from the bye-standers' report, was prostrate, with the face to the ground. She was picked up immediately, and brought to the hospital in a state of extreme collapse, with the skin cold, the face pale, and the lips white. A graze of the integuments was found in the loins, but no fracture could be felt. When put to bed she vomited; stimuli were given but she never rallied; and at the expiration of between two and three hours she expired.

*Dissection.* On opening the abdomen, about a pint of bloody fluid was found in it, but no rupture of any large vessel could be detected. The principal extravasation appeared to be in the right renal, lumbar, and iliac regions. The synchondrosis pelvis on the right side was separated, and near the acetabulum, but apparently not into it, the os pubis was broken. The latter fracture extended into the foramen ovale. The transverse processes of the 4th and 5th lumbar vertebræ were broken across at their roots.

On opening the heart the foramen ovale at its most inferior part was found to be pervious, and presented a circular aperture, the bore of which was full as large as, if not larger than a goose's quill. The ductus arteriosus was closed at its aortic end, but at its origin from the pulmonary artery, it presented a small blind foramen.

Here we see the foramen ovale pervious, and the opening a large one too, without producing apparently any inconvenience, or indeed any symptom to mark its presence. We think there can be little doubt that in early life at all events, and probably also in mature age, the open foramen ovale is not of that consequence physiologists would lead us to believe. When we say the open foramen ovale, we mean that malformation only, and not combined with other disease or disorder of the heart. When the pervious state of the foramen is accompanied with enlargement and flabbiness of the organ of the circulation it becomes a much more serious matter, as the following case will amply prove.

*Case* 2. A woman beyond the middle age, who had been in the house more than once for obstinate ulcers of the leg, was received again on the 1st of April for a return, or rather the continuance of her old complaint. This person's appearance was rather remarkable. There was a high flush upon the cheeks, and a kind of scurfiness or coarseness of the skin, giving a disagreeable cast to the countenance. The surface of the body generally was of a very peculiar hue, neither bright red, nor yet purple, but something between them, and nearer a salmon colour than any thing else to which we can compare it. It struck one, on looking at her, as that of a person whose blood, to use a vulgar phrase, is in a very bad state. We thought when we saw her that the foramen ovale might be open, but on questioning her, she stated that the colouration of the skin had only been present for five or six years. We made at this time no further enquiries, nor did we ascertain whether any or what symptoms existed to indicate disease or malformation of the heart.

On the evening of the first of May, she was seized with what seemed to be erysipelas of the leg, and so violent was the attack, and so rapid its termination, that by midnight of the 2nd, she was dead. Since her re-admission, we had not seen her, and the following were the appearances presented on dissection, which took place upon the 4th.

The left lower extremity was in a state of gangrene. The whole back and outside of the thigh presented one immense vesication filled with dark, port wine coloured serum. About the leg there were many vesications, and the limb was generally of a dusky mottled appearance. The superficial veins throughout the body were quite as distinct as if they had been injected, the circumstance evidently depending on the stasis of dark blood within them. A colour still remained upon the cheeks, a very unusual occurrence indeed. The body externally was not very fat, but on opening the abdomen, the omentum, mesocolon, and mesentery presented one sheet of continuous solid adeps, in which nothing like the vessels of the parts could be distinguished. The deposition of fat was equally great in various other parts of the abdominal cavity. The spleen was half fluid—the liver soft and lacerable with the slightest force—the kidneys in a similar condition. The os tincæ was plugged up with gelatinous matter; the uterus presented in its substance, immediately beneath its peritoneal coat, one or more tubercular patches, a little larger than peas.

A good deal of fat was found in the anterior mediastinum and investing the pericardium. On cutting into the latter, the heart was seen to be large in dimensions, but extremely flabby. Much fat was deposited upon it externally, especially in the line of the auriculo-ventricular sulcus. The parietes of the organ were so thin and rotten, that the finger was pushed through them with the greatest ease. The foramen ovale was open, and the aperture was nearly as large as a six-pence. It would have been larger but for a crescentic fold of membrane which bounded it above. The Eustachian valve was very distinct.

Both the lining membrane of the heart and of all the large vessels that were opened, was stained of a deep mahogany colour by the blood, which was universally fluid.

The cavity of the chest was small, and the lungs surprisingly so. Instead of presenting the healthy light grey colour, they were of a deep æruginous tint, compact and dense. They crepitated, however, and shewed nothing like a trace of inflammation. We should say that in point of size, the lungs were one third smaller than they should be in proportion to the body.

The breasts were very large, and the glandular part was almost entirely converted into fat.

Several points in the foregoing case would be worth consideration, did our space or inclination permit us to venture on a sea of conjectures. It is remarkable, however, that although the foramen must always have been open, the symptom dependent upon it, we mean the coloration of the skin, should only have existed for five or six years. Are we to suppose that till then the heart itself was sound, and performed its functions tolerably well, despite of the malformation in question; but that then, and not before, the enlargement of the cavities and thinning of the walls of the circulating organ, made a scale in the balance of the circulation kick the beam? Our readers may form what conjectures they please upon the subject, but for our own parts, we are disposed to favour the supposition. The state of the lungs also merits remark. Was their miniature size a congenital defect, or was it the result of the state of the heart, and the consequent imperfection of their own functions? Muscles waste when little used, and why may not the lungs?

II. TREATMENT OF NÆVI.

CASE 1. *Subcutaneous Nævus of the Lower Eye-lid—Excision performed.*
A child, about 10 months old, was brought into the operating theatre on the 23d of March, with a subcutaneous nævus, the size of a nut, on the lower lid of the right eye, not mounting quite so high as the tarsal cartilage. Mr. Brodie excised it, removing it, together with the skin over it, by two semi-elliptical incisions. One or two vessels bled smartly and required to be tied, when the wound was dressed with lint and sticking plaster, and the child sent home. The wound healed without any thing untoward or unusual, and the cure was effected without ectropion of the lid.

CASE 2. *Subcutaneous Nævus of the Upper Lid—Modification of the Ligature.*
The child was four months old, and the nævus, about the size of a large Spanish nut, was situate on the upper palpebra of the left eye, and passed down to the margin of the tarsal cartilage, which was overlapped by the little tumour, but did not appear to be implicated with its origin. Mr. Keate introduced a curved needle armed with a double ligature under the base of the tumour, carefully avoiding the fibrous layer that forms the lid. The needle was passed quite through transversely, from side to side, and then a second was passed with the same precaution from before backwards, exactly at right angles with the former which it crossed. The needles were then cut away and the ligatures remained. These were severally tied at the corners, so that there were four knots, and the last tightened the rest, in a manner that we need not stop to explain. It was many days before the nævus sloughed away, and the ligatures required tightening several times before the separation was effected. We saw the child lately, and no ectropion of the lid had taken place.

CASE 3. *Cutaneous Nævus of the Back—Excision.*
On the 23d of March, immediately after the case first detailed had been disposed of, a child younger still, and healthy in appearance, was operated on by Mr. Keate. The nævus was irregular in shape, of considerable dimensions, placed over the dorsum of the right scapula, and evidently not seated beneath the skin, but in the substance of the cutis itself. It rose up abruptly at its margin from the surface of the neighbouring skin, and was covered by a thin layer of cuticle. Mr. Keate excised the tumour by including it between two semi-elliptical incisions, and then dissecting it away. An artery of some little size distinctly entered at one corner of the nævus, and a vein apparently issued from it at the other. The artery was taken up, and the wound dressed with sticking-plaster and compress.

The wound did not heal by the first intention; a smart attack of erysipelas succeeded, spread over a considerable part of the back, the whole of the arm, and other parts of the trunk, and, finally, it was two or three weeks before the child and the wound were well. We saw it, however, on the 21st of April, when it looked hearty, and was perfectly recovered.

III. INJURIES OF THE HEAD.

CASE 1. *Scalp Wound. Some Symptoms like Delirium Tremens.*
Thomas Bevan, ætatis 27, was admitted in the evening of the 26th of April, under the care of Mr. Keate. This man was a servant at a distiller's, and had been so for 14 months. During that time, having had a *carte blanche* to the spirits, he was tolerably often drunk, and during that time, also, but never before, he had been subject to what seemed to be epileptic fits. On the evening of the 26th, whilst intoxicated, he fell against the bannisters, and cut his forehead. The wound was perpendicular, small, and shallow, and the bone was not exposed. When admitted it was hard to say whether he was suffering from the effects of his potations, or the wound upon the head. The latter was dressed, and the patient put to bed. Next morning he had recovered his senses so far as to tell how the accident happened, and confess to his habits of inebriation. He had early in the morning been seized with some symptoms of extreme nervousness and irritability for which he had been bled with good effect. On the 29th he was free from any urgent symptoms, and the wound was all but healed; but still there was a degree of irritability about him indicative of the life he had led, and the kind of action that a slight cause perhaps might set up. On the 10th of May the wound had been healed for a week or more, and the patient was free from complaint.

CASE II. *Scalp Wound denuding the Cranium—Urgent Symptoms—Bleeding—Erysipelas.*

George Foley, ætatis 32, admitted April 28th, under the care of Mr. Keate.

He had just fallen with a scaffolding in Grosvenor Place, from a height of 40 or 50 feet upon the ground, and received an injury of the head, with a superficial wound and contusion of the knee-joint. On the right side of the head, a little above and anterior to the ear, was a scalp-wound, jagged, and leading down not only to exposed pericranium but bare bone. There was no fracture. He had been stunned by the fall, and at the time of his admission was not recovered. The pupils were sluggish; the man restless, fidgetty, and unable to answer questions; the pulse was but little affected; he had had no vomiting. The portio dura on the side of the injury appeared to be partially paralysed, for the mouth was decidedly drawn towards the left.

In the evening the pulse got up and he was bled to ten or twelve ounces. Next day he was greatly recovered, so much so indeed as to be able to give a very good account of the manner in which the accident occurred. He had had no sickness and passed a good night; the pulse was risen, not very hard nor quick, but still it was risen; the pupils acted, but were somewhat contracted; the paralysis of the right portio dura was lessened; there was no head-ache. The wound had been dressed, the neighbouring part of the head shaved, and cold lotion constantly applied. He had also been purged by the house-medicine, and took salines with the sulphate of magnesia every four hours. In the afternoon of the 29th he was flushed in the face, and the pulse had got up; in the evening he was bled to ten or twelve ounces.

30th. Not so well to-day. The patient is drowsy and heavy—has pain in the site of the wound—pupils more inclined to be contracted—still some distortion of the mouth—skin warm—pulse not very hard nor quick, but full—bowels not opened—no nausea nor shivering. The wound looks foul and is covered with a poultice; the blood abstracted last night is not inflamed.

*Rep. haust. salin. c. Magnes. sulph. addendo vin. ant. tart. m. xxx.*

Next day there was little alteration, save that the pain in the head was rather less, and the tongue was more moist. He had had no rigor. In the evening he was bled to ℥x. and on the 2d, presented very urgent symptoms. He had passed a bad night—there was much head-ache—extreme drowsiness—face flushed—skin very hot—pulse quick and bounding—scalp tender about the wound and tumid. There had been a kind of chill succeeded by heat, but no distinct rigor. He was bled to ℥viij. and very soon afterwards the blood shewed the buffy coat. In a couple of hours or thereabouts, the vein was re-opened and 33 ounces of blood abstracted. This had a marked effect upon the pulse; the skin grew pale; the heat diminished; and a copious perspiration succeeded. At 5, p.m. re-action had not set in; the pulse was soft, the patient in a tranquil state.

*May 3d.* An erysipelatous blush has shewn itself upon the forehead, attended with tumefaction of the subcutaneous parts of the scalp. The tongue and skin are moist—pulse soft—headache not severe—restlessness trifling.

*Pot. subcarb.* ℈j. *aq. distill.* ℥iss. *succ. limon.* ℥ss. *4tis horis.*

On the 4th, the erysipelas had extended down the forehead to the bridge of the nose and cheeks. The tint was very pale, owing, no doubt, to the active bleeding practised on the 3d. The symptoms were now as favourable as could be wished; skin moist—pulse soft—no delirium—head-ache slight—tumefaction of scalp not great. On the 5th he complained of nothing but feeling low, and every thing indicating the exhibition of bark, it was ordered with the liquor ammoniæ acetatis. The scalp-wound looked clean, but the portion of bone was still exposed.

℞. *Liq. amm. acet.* ʒij —*aq. distill.* ʒvj.—*decoct. cinch.* ℥ss. *4tis horis.*

The bark agreed well, the erysipelas rapidly arrived at its acmé and scaled, the feverish symptoms quickly passed away, and on the 10th the patient complained of nothing except hunger. Stimulating diet, of course, was prohibited, and every thing at the date of this report, 13th of May, would seem to augur rapid convalescence.

This was certainly at one time an urgent case, and the combination of symptoms on the 2d inspired a not ill-founded dread of mischief brewing within the skull. It will be seen from the report that, previous to these unfavourable symptoms occurring, the patient had experienced a chill, but no distinct rigor. This we consider an important circumstance, and one that will generally enable us to predict that erysipelas is impending, rather than the formation of matter. In the cases of the formation of pus upon the dura mater the rigor, as far as we have witnessed, is generally very severe, and exceedingly unlike a mere chill. When erysipelas, on the contrary, is making its approach, the shivering is seldom of much severity, and indeed is often entirely overlooked by all parties. This, we should say, is at times an important criterion in these dangerous and frequently obscure cases. It may be asked whether, under these circumstances, a bleeding to 33 ounces was indicated? We believe that it was; for, setting aside the doubt that must necessarily exist as to the positive nature of the case, it seems that the active depletion almost "put out" the erysipelas, and modified it greatly at all events. Stout, healthy men, attacked with erysipelas after scalp-wound, will bear both bleeding and depletion a thousand times better than any other persons, at least in hospital, labouring under that disease. We remember witnessing the case of a soldier who was treated throughout by very active phlebotomy indeed, and recovered well. If speedy and decided buff upon the blood, with strong pulse, hot skin, and loaded tongue, are to be considered as indications for blood-letting, they were all present in this instance.

The next case to which we shall advert is one of a more melancholy character.

CASE III. *Compound Fracture of the Skull—Abscess in the Brain.*

Henry James, 11 years of age, was cleaning a horse on the 28th of April, when he received a kick on the back of the head, which produced a wound, and instantly stunned him. It was said that in the course of a little while he recovered, but quickly relapsed into his former insensibility. About an hour after the accident he was brought into the hospital, previously to which he was sick and vomited. In half an hour after his admission we saw him.

He lay tolerably quiet, when not disturbed, without any stertor or convulsion. On asking if he had any pain, and rousing him from his lethargic state, he stared, but made no reply; on attempting to look at the wound he grew quite unmanageable, rolling about in the bed and trying to thrust away the hand. The pupils were dilated—the pulse by no means devoid of force—the skin warm. Just above the spine of the occipital bone and a little to the left of it was a transverse wound of the scalp, about an inch in length, jagged, and leading to the bone, which was broken, depressed to the depth of a line, or rather more, and apparently splintered. The fracture, as far as we could judge, was just above the left lateral sinus. The nurse informed us that, just before we saw him, he had had convulsions, but they had, by this time, completely passed away. He had not been bled.

The back part of the scalp was shaved, the wound was dressed, and, in the evening, he was bled to ℥iv. and took a powder, consisting of three grains of calomel and three of the pulvis antimonialis.

29th. He vomited again last evening, but has had no return of the convulsions. To-day he is much more sensible, being able to answer questions rationally. The pupils act—the pulse is quick and rather full—tongue moist—skin hot. A poultice has been applied to the wound, around which there is much tumefaction. During the early part of the 30th he made no complaint, but, towards the afternoon, he suffered from pain in the head—the skin was warm—the pulse a little quick and sharp—the tongue whitish, but moist. He had had no rigor nor vomiting. In the evening he was bled to 5 ozs. and soon afterwards the blood was cupped and buffed. On the 1st of May the pulse had some jerk and the skin was warm, but the pain in the head was diminished. The bowels were freely opened, and neither rigor nor vomiting had occurred.

*May 2d.* Complains of much headache; he is drowsy; the tongue white and moist; pulse pretty quiet; pupils a little dilated; bowels open; no rigor. A poultice is kept to the wound, which looks dirty and sloughy, and has some tumefaction around it. He is taking salines, with ℈j. of the sulphate of magnesia.

Next day he was much the same, drowsy still and heavy, with the pupils apparently little affected, but a good deal of pain complained of in the temples. Eight leeches were applied in the evening, but the pain in the forehead was still severe upon the 4th; the pulse was 80, with some force; the bowels open. He was bled that evening to three ounces, and, on the 5th, was worse rather than better. He had constant, but not violent pain in the forehead, drowsiness and malaise, with a foul appearance of the wound and surrounding tumefaction. Under these circumstances, Mr. Keate enlarged the wound and examined the state of the fracture. It was about an inch or two in length, with a little fissure extending downwards, but not far. The superior portion of bone appeared to be depressed below the inferior, but the depression, as we stated before, was not above a line or two in depth. We thought, however, that the tables appeared to be separated as far as they could be in so young a subject. Mr. Keate did not trephine, but applied lint between the edges of the wound, and over that a poultice and lotion.

On the 6th, the child was still heavy, and to us appeared rather incoherent. The pulse was 84, full and sharp; the pain was still present in the brow. In the evening he was bled again to 3 ozs. but the next day brought no perceptible amelioration, nor, indeed, any material change in the symptoms. The pain in the forehead continued—the pupils appeared to be a little dilated, and the patient was frequently complaining of weakness and soreness of the right eye—he was drowsy—loth to be disturbed, but yet answering questions when spoken to—and had had in the morning, according to the mother's report, a shivering. Of the latter fact, however, we could by no means obtain a satisfactory account. On the 8th, he was still in much the same condition, but, during the night, he was suddenly seized with an epileptic fit, from which he recovered, but died an hour or two after midnight.

*Dissection.* The scalp was reflected from the calvarium in the usual way, when the whole of the fracture was exposed. Its extent and form were found to correspond with what we have already stated, and the pericranium, for some little distance around, was detached from the surface of the bone. On removing the calvarium, the dura mater, as far as it was seen, was intact, and on cutting round the membrane, and turning it back, the surface of the brain was equally uninjured. The hemispheres were sliced down to the level of the lateral ventricles, when the left was found filled with a mixture of serum and pus. The posterior horn was discovered, on prosecuting the examination, to open into an abscess, the size of a walnut, in the posterior part of the left hemisphere, exactly opposite the fracture. The right ventricle contained also purulent fluid, but not to so great an extent as the left. The brain was removed, and the state of the fracture internally observed. The inner table had been separated in some degree from the outer, and the depression internal to the skull was greater in consequence, than that which existed without. The sharp edge had pierced the dura mater in one spot, and apparently pierced the brain also, at least it was here that the abscess in the latter had begun. Between the dura mater and the bone was a very slight extravasation of blood, but no pus. The membrane, however, at this spot was inflamed. Some lymph was found at the basis of the brain and about the decussation of the optic nerves.

In consequence of the reluctance expressed by the friends, none of the other cavities were opened.

This case is one of much practical importance, for it serves to illustrate the truth of the opinion advanced by Sir Astley Cooper and maintained by Mr. Brodie, respecting the dangerous nature of compound fractures of the skull. We suppose there can be little doubt that the spicula of bone, piercing through the dura mater and constantly pressing on, if not actually entering the substance of the brain, was a permanent source of irritation to the organ. It was a source of irritation very likely to excite inflammation and suppuration in the substance of the brain, and, cæteris paribus, more likely than its removal by the trephine would have been, to lead to those fatal results. The symptoms, during the last five or six days of the boy's existence, were clearly those of inflammation rather than compression. The constant pain in the forehead, the sense of weakness or pain in the eyes, more especially the right, the pulse above par, but yet not much —were symptoms indicative of the insidious mischief going on within. Will the absence of pus upon the membranes account for the want of any distinct rigor?

